# Conditions of Success in Ovariotomy

**Published:** 1885-03

**Authors:** Robert Battey

**Affiliations:** Rome, Ga.


VOL. 2. MARCH, 1885. NO. 1.

### Original Communications.

### CONDITIONS OF SUCCESS IN OVARIOTOMY.

BY ROBERT BATTEY, M. D., ROME, GA.

The extraordinary results in ovariotomy which have been obtained in Great Britain within the past three years—seventy-three consecutive operations in the hands of one surgeon, and seventy-six in the hands of another, without a death—are well calculated to excite both astonishment and admiration. When we contrast these figures with the published results obtained by American operators, exhibiting a mortality, still, of twenty and twenty-five per cent, it certainly is not an impertinent inquiry. Why is this?

It may be soothing to our national pride to comfort ourselves with the belief that our cases are more grave than those encountered by our British brethren, or that they select their cases whilst we do not. Such assumption on our part, does not seem to me, to accord with the facts as observed by those who have had opportunity. Moreover, the tendency of this self-complacency is not favorable to that careful scrutiny of our own methods which would tend to improve them, and to lessen the wide disparity in the statistics. It is not my purpose to enter upon a critical comparison of the British and American methods in ovariotomy, but simply to consider, briefly, what I conceive to be some of the conditions of success, and more especially to draw forth the views of others whose larger experience enables them to speak with an authority upon this subject far beyond my own. I shall treat the conditions of success in the order in which my own observation has seemed to indicate their relative importance.

FIRST—EXPERIENCE AND SKILL IN THE OPERATOR.

Little need be said under this heading. The proposition commends itself at once to any reflecting mind. Practice makes perfect, in ovariotomy as in any other handicraft. The statistics of the great operators show a steadily increasing ratio of success, which is not to be explained, fully, by improvement in methods, but is largely due to the skill and dexterity which grows of much practice. If the best results are to be obtained in America, ovariotomy must be put into the hands of the few, and the general practitioner must forego the ambition to swing, here and there, an occasional scalp to his girdle.

SECOND—CLEAN HANDS AND APPLIANCES.

I can bear testimony to the painful fact that I have, myself, consigned a number of cases to a premature grave by insufficient attention to the careful cleaning of the finger-nails, knives, forceps, ecraseur chains, and sponges. In no respect has recent improvement in the operation been more marked than in the superlative estimate of the value of extreme care upon this point in the minds of skilled operators. The chain ecraseur is so complicated an instrument that its thorough cleansing is laborious and difficult, and no case can be positively assured. For this reason alone it should be rigidly excluded from the ovariotomy case. Before operating, the hands and forearms should be carefully and repeatedly washed in warm water, using a large nail brush and a good detergent soap, especial care being given to the nails, and hands well bathed in carbolic solution (1 to 40) or other antiseptic. After operating, my instruments and sponges are cleansed with warm water and strong soap; instruments are anointed well with carbolic cerate, and sponges placed in a jar with solution of sodic carbonate. Upon the following day the instruments are again gone over with hot water and soap, using a stiff brush to reach every crevice, anointed afresh with carbolic cerate and laid away. The sponges are washed out and replaced in fresh sodic solution daily for a week, when they are transferred to solution carbolic acid (1 to 40) in which they are allowed to remain until needed for use. Preparatory to the next operation, all instruments are again subjected to the hot water, soap and brush, and covered with the carbolic solution for an hour or two before they are needed for use. It is only after much labor and care to remove completely all traces of sand and dirt that new sponges can be fitted for ovariotomy. The ovariotomist should not be in attendance upon cases of puerperal fever, scarlatina, diphtheria, erysipelas, or other malady which would endanger the safety of the surgical patient. It were better that he should be a specialist, giving himself, as far as possible, to his special work.

### THIRD—CLEAN APARTMENT AND BEDDING.

The apartment for ovariotomy ought to be a special ward devoted to this purpose alone; at least two such apartments are required, if more than an occasional case is to be dealt with. The upper floor, in an elevated and airy location, is desirable, having free access of pure air and sunlight. The woodwork and floor should be well painted, and the paint renewed each year. The walls should be limed, not papered or frescoed, and the liming should be renewed for each case. The furnishing should be scanty and simple, without carpet, curtains or upholstered lounge. Rubber cloth may be spread from the door to bed and fireplace, to deaden sound in walking. The windows are darkened by slatted blinds. The bed should be fresh, scrupulously clean and thoroughly comfortable; feathers excluded except in pillows; brass bedsteads fitted with the Hartford woven-wire are most desirable, though the iron bedstead, with a fresh coat of paint each season, answers an excellent purpose. I have found the best mattress to be carefully made of hackled shucks and carded cotton. It should be replaced with a new one once a year, and at any time when it becomes soiled, or has been used for a suppurative or drained case. The covering should consist of sheets, woolen blankets and white counterpane, all of which are to be well washed when needed, and also at each emptying of the apartment. The supply of linen should be ample, and care taken that soiled bedding, clothing, bed pans and wash waters are promptly removed. At each emptying of the ward, the bedding is removed, the walls freshly limed, the floor and woodwork well scrubbed, using strong soap and carbolic acid, and continuously aired until again needed for use.

FOURTH—PURE ATMOSPHERE AND FREE VENTILATION.

The evil results of hospitalism are largely due to atmospheric contamination. The vitiated atmosphere of large cities is unfavorable to the healthful repair of surgical wounds, and especially so when admitted to the opened abdominal cavity. With like skill and facilities for operation, after-care and nursing, the best results are to be expected in a pure, country atmosphere. It must, however, always be difficult to secure the service of a skilled surgeon, experienced ovariotomy nurse, and the advantages of well appointed ovariotomy wards, remote from the great centres of population, where the patient may enjoy the invigorating influence of a pure mountain air. To avoid this difficulty recourse is had to the suburbs of cities and to the upper floors of buildings in elevated urban localities, with surroundings as salubrious as circumstances will admit. I believe it to be a fatal mistake to do ovariotomy at the homes of patients, in unsuitable apartments, with insufficient appliances, inexperienced after-attention, ignorant nurses, and in a noisy household, however favorable may be the outward surroundings.

FIFTH—THOROUGH CLEANSING OF THE ABDOMEN ; DRAINAGE.

I believe it to be a well established fact now that the great danger to be encountered in ovariotomy is sepsis, and not, as was formerly believed, peritonitis. Septic poisoning may arise either from portions of the cystic contents having escaped into the abdominal cavity, or from the decomposition of bloody serum which has been but imperfectly removed. A most important security against this great source of mortality is to be found in the careful arrest of all bleeding, and the thorough cleansing of the abdominal cavity. Dr. Keith, of Edinburgh, was one of the first to recognize this great fact, and his extraordinary success is doubtless largely due to his careful attention to it.

Cases will occasionally occur where, in spite of all our care, by rupture of the cyst or otherwise, portions of its noxious contents escape into the abdomen, pass about amongst the viscera, and so infect the cavity that we cannot be quite sure that the most diligent sponging has fully effected its object. Again, the oozing points from broken down adhesions may be so very numerous as to defy our attempts to completely arrest the seeping, or the peritoneum may be pouring out serum copiously, or shock may be so profound as to compel us to desist from our efforts at complete toilet of the cavity. In either of these contingencies a valuable resource is to be found in the drainage tube, which, with proper antiseptic precautions, conveys away harmlessly the offending liquid.

SIXTH—SKILLED NURSING AND QUIETUDE.

These essential requisites to the well-doing of any surgical operation are especially important after ovariotomy, and cannot well be secured at the home of the patient. Experience in this class of cases can alone fit the nurse for her duties; for the principles which are to guide her in the handling and dieting of the patient are special and peculiar, and are not to be acquired in the course of ordinary nursing, however protracted. The nurse should be expert with the catheter and syringe, so as to be enabled to use these instruments properly without unnecessary disturbance. She ought also to be dextrous in changing the clothing with the least amount of moving of the patient, and when change of position is desirable, she should be able to effect it without giving pain.

SEVENTH—EARLY OPERATION.

Until quite recently the opinion amongst operators was well nigh universal that long carrying of an ovarian tumor and well marked emaciation produced a certain change in the peritoneum and a surgical tolerance which were especially favorable to recovery. This opinion rested wholly upon the experience of ovariotomists. Recently, however, since the introduction of the antiseptic methods in ovariotomy, the teaching of experience has completely reversed this rule. When an ovarian tumor is of recent origin, of small size, free of adhesions to other organs or structures, having but little solid material and no malignant deposits, and the general health not broken down, it is reasonable to expect the best results; and this expectation is fully justified by later observations.

EIGHTH—INTERNAL TREATMENT OF THE PEDICLE BY LIGATURE.

The treatment of the pedicle in ovariotomy has long been the subject of discussion and always esteemed important. Even a brief consideration of the various methods which have been practiced, and the reasons for their advocacy would consume time unnecessarily. With the later improvements in the operation, which have given such excellent results, it has been found desirable to so treat the pedicle as to admit of the prompt and complete closure of the thoroughly cleansed abdominal cavity. Secondly, to leave the pelvic organs in situ and at rest, avoiding all dragging upon the uterus. Thirdly, to secure complete immunity from secondary hemorrhage from the cut surface. Fourthly, to leave no foreign body calculated to excite undue inflammation, suppuration, or decomposition. Experience has shown that all of these desirable points are satisfactorily secured by the use of the carbolized silk ligature, well tied and cut short.

NINTH—ANTISEPTIC SOLUTIONS AND SPRAY.

Much difference of opinion still exists as to the value of carbolic acid, and other antiseptic solutions, in ovariotomy. The results obtained by Dr. Bancock, in London, and Mr. Lawson Tait, in Birmingham, seem to show conclusively that the use of these solutions is not indispensable to the attainment of the best success. They have both shown by their work that scrupulous attention given to the cleansing of hands, instruments and sponges, not only prior to operating, but frequently during the progress of the operation, is sufficient. The frequent removal of the blood from hands and implements appears to protect the abdomen from septic influences.

Dr. Keith, of Edinburgh, whose painstaking and conscientious work in this department of surgery has never been excelled, discards the use of the spray now, but retains the carbolic solutions for his hands, instruments and sponges. He discards the spray, not upon the ground that it is useless, but because he esteems it positively injurious, and even fatal, in its effects upon certain patients. He has also suffered seriously in his own person whilst using it. It was the practice in Great Britain to employ in the atomizer a carbolic solution of one to twenty, and the spray was directed strongly upon the wound and down into the abdominal cavity whilst operating. In many cases the renal secretion was very much diminished, and in a few entirely suppressed, by the acid circulating in the blood.

In view of these facts it may be asked, ought not the antiseptics to be entirely abandoned in ovariotomy? From the standpoint of my own observation and experience, I am inclined to answer, no. The very great improvement in the statistics of ovariotomy which followed at once upon the introduction of the carbolic spray and solutions, are too recent and too striking to be either forgotten or ignored. In my own work I have found the solution of one to twenty too strong for my hands, and reduced it to the ratio of one to forty. The same solution I use in the atomizer and this reduced still further by the condensed steam, gives a spray of strength probably not greater than one to eighty. The spray is thrown from a distance across the abdomen and not down into the cavity. In no instance have I had reason to suspect the least harm to my patients from such use of carbolic acid. To the criticism that carbolic solutions weaker than one to twenty have been shown in the laboratory to be impotent for the destruction of bacteria, I answer that I am seeking by its use only the restoration of my patients to health, and the mortality in my hands since its use has dropped from twenty-five per cent to zero. This, for me, is sufficient reason for the continuance of the method, and for the rejection of all other substitutes until such time as more complete demonstrations shall place a clearer light before me.

